# Mitochondrial Disease in Autism Spectrum Disorder Patients: A Cohort Analysis

**DOI:** 10.1371/journal.pone.0003815

**Published:** 2008-11-26

**Authors:** Jacqueline R. Weissman, Richard I. Kelley, Margaret L. Bauman, Bruce H. Cohen, Katherine F. Murray, Rebecca L. Mitchell, Rebecca L. Kern, Marvin R. Natowicz

**Affiliations:** 1 Cleveland Clinic Lerner College of Medicine, Cleveland Clinic, Cleveland, Ohio, United States of America; 2 Department of Pediatrics, Johns Hopkins University Medical Center and Division of Metabolism, Kennedy Krieger Institute, Baltimore, Maryland, United States of America; 3 Department of Pediatrics and Learning and Developmental Disabilities Evaluation and Rehabilitation Services (LADDERS), Massachusetts General Hospital, Boston, Massachusetts, United States of America; 4 Neurological Institute and Pediatrics Institute, Cleveland Clinic, Cleveland, Ohio, United States of America; 5 Genomic Medicine Institute, Cleveland Clinic, Cleveland, Ohio, United States of America; 6 Pathology and Laboratory Medicine Institute, Cleveland Clinic, Cleveland, Ohio, United States of America; National Institutes of Health, United States of America

## Abstract

**Background:**

Previous reports indicate an association between autism spectrum disorders (ASD) and disorders of mitochondrial oxidative phosphorylation. One study suggested that children with both diagnoses are clinically indistinguishable from children with idiopathic autism. There are, however, no detailed analyses of the clinical and laboratory findings in a large cohort of these children. Therefore, we undertook a comprehensive review of patients with ASD and a mitochondrial disorder.

**Methodology/Principal Findings:**

We reviewed medical records of 25 patients with a primary diagnosis of ASD by DSM-IV-TR criteria, later determined to have enzyme- or mutation-defined mitochondrial electron transport chain (ETC) dysfunction. Twenty-four of 25 patients had one or more major clinical abnormalities uncommon in idiopathic autism. Twenty-one patients had histories of significant non-neurological medical problems. Nineteen patients exhibited constitutional symptoms, especially excessive fatigability. Fifteen patients had abnormal neurological findings. Unusual developmental phenotypes included marked delay in early gross motor milestones (32%) and unusual patterns of regression (40%). Levels of blood lactate, plasma alanine, and serum ALT and/or AST were increased at least once in 76%, 36%, and 52% of patients, respectively. The most common ETC disorders were deficiencies of complex I (64%) and complex III (20%). Two patients had rare mtDNA mutations of likely pathogenicity.

**Conclusions/Significance:**

Although all patients' initial diagnosis was idiopathic autism, careful clinical and biochemical assessment identified clinical findings that differentiated them from children with idiopathic autism. These and prior data suggest a disturbance of mitochondrial energy production as an underlying pathophysiological mechanism in a subset of individuals with autism.

## Introduction

Autism spectrum disorders (ASD) are neurodevelopmental disorders characterized by impaired social interaction and communication, as well as isolated interests and repetitive or stereotyped behaviors [1]. ASDs pose a significant burden to affected individuals, their families and society. This burden comes both from the debilitating and lifelong nature of ASDs and from their prevalence. It is now estimated that about one out of every 166 children is affected with ASD [2]. Most cases are idiopathic, although there are many uncommon or rare genetic and metabolic causes of autism that are increasingly recognized [3]–[5].

In 1998, Lombard postulated mitochondrial dysfunction as a cause of autism [6]. Prior and subsequent case reports provided biochemical data indicating perturbation of mitochondrial energy metabolism in some individuals with ASD [7]–[12], including mtDNA mutations in several [10], [13], [14]. Recently, Oliveira and colleagues published a population-based survey of school-age children with ASD. They found that 7% of those who were fully tested met criteria for definite mitochondrial respiratory chain disorders and were also clinically indistinguishable from other children with ASD [15]. This work is notable because it suggests that mitochondrial disorders of energy production may be present in a substantial percentage of children with ASD. To better describe the clinical spectrum of children with ‘mitochondrial autism’, we undertook a chart review of the biochemical, genetic, and histopathological findings in 25 patients with ASD who had unequivocal evidence of a disorder of oxidative phosphorylation.

## Methods

### Subject Determination

We reviewed medical records of 25 children with a diagnosis of ASD according to DSM-IV-TR criteria [16] and evidence of a disorder of mitochondrial energy metabolism evaluated by one of our investigators at Cleveland Clinic, Massachusetts General Hospital/LADDERS Program, or Kennedy Krieger Institute. The study was approved by the Institutional Review Boards (IRBs) of these institutions and is in accord with the principles of the Declaration of Helsinki. The IRBs determined that this chart review study met criteria for waiver of consent.

Children were referred for genetic and/or metabolic evaluation of autism but not specifically for evaluation for mitochondrial disease. Children with known chromosomal or monogenic disorders were excluded. All patients met criteria for probable or definite mitochondrial disease by both the Modified Walker Criteria and the Mitochondrial Disease Criteria (MDC) [17], [18]. All patients also had either: (1) <20% activity of one or more muscle or fibroblast electron transport chain (ETC) activities; (2) <2 standard deviations below the control mean of one or more ETC complexes by polarographic analysis; (3) a mitochondrial DNA sequence variant of probable pathogenicity plus biochemical evidence of mitochondrial dysfunction; or (4) clinical and biochemical data consistent with a mitochondrial disorder and a sib with enzyme-defined ETC dysfunction. One patient was reported earlier [12].

### Clinical and Biochemical Data

Medical records of each patient were independently reviewed by at least two members of the study team. Growth parameters were evaluated using the ABase software and North American pediatric reference values [19]. Institution specific reference intervals were used for assessments of blood lactate and pyruvate, plasma alanine, urinary organic acids, CK, AST, and ALT. Biochemical evidence of mitochondrial ETC dysfunction included increased blood lactate and pyruvate levels, elevated plasma alanine level, and increased urinary levels of Krebs cycle intermediates or 3-methylglutaconate.

### Pathology and Enzyme Data

Twenty-three of the 25 children underwent quadriceps muscle biopsies, 11 had skin biopsies, and one had a liver biopsy. Three patients had sibs with muscle biopsies. Muscle ETC determinations were performed at either Horizon Molecular Medicine, LLC, Atlanta [20], [21] or the Center for Inherited Disorders of Energy Metabolism (CIDEM) Lab, Cleveland [22]. Muscle biopsy specimens were either snap frozen then shipped for ETC determination in homogenates or muscle mitochondria were isolated from fresh muscle biopsy specimens with aliquots frozen for subsequent determination of ETC activities. Functional (polarographic) analyses of oxidative phosphorylation using freshly isolated muscle mitochondria were performed at CIDEM [22]. ETC determinations of skin fibroblasts were done at CIDEM [23] or Mayo Laboratories. Fibroblast lactate and pyruvate measurements were performed at The Hospital for Sick Children, Toronto [24]. ETC activities were normalized to the mitochondrial marker enzyme citrate synthase, or if citrate synthase activity was not assayed, the percent of the mean of controls was used.

### Mitochondrial DNA (mtDNA) Mutation Data

Sixteen of 25 children underwent analyses for selected mitochondrial mutations and 11 of 25 patients had mtDNA mutation analysis by dHPLC of the entire mitochondrial genome at Transgenomic, Inc, Omaha [25]. Each DNA sequence variant was evaluated for pathogenicity by a search of the MITOMAP and mtDB–Human Mitochondrial Genome databases [26], [27], PubMed, and compendia of mtDNA mutations including guidelines for determination of pathogenicity [28], [29].

## Results

### Clinical Characteristics of Subjects (Table 1; Tables S1, S2, S3)

Twenty-five patients–13 males and 12 females ages 2–20 years–were included in this analysis; 11 patients met DSM-IV-TR criteria for autistic disorder and 14 met DSM-IV-TR criteria for PDD-NOS. Twenty-one patients had definite mitochondrial disease and four had probable mitochondrial disease by the Modified Walker Criteria. Eleven patients met criteria for definite mitochondrial disease and 14 met criteria for probable disease using the MDC.

**Table 1:** Medical History and Physical Examination.

	Number of patients	Percent of patients
**Multiple gestation birth**	8	32
**Prenatal or perinatal complications**	13	52
**Cardiovascular abnormality**	7	28
**GI dysfunction**	16	64
**GI dysfunction other than reflux or constipation**	5	20
**Hematologic abnormality**	2	8
**Endocrine dysfunction**	2	8
**Renal dysfunction**	2	8
**Growth retardation (<2 SD for weight and/or height)**	5	20
**Microcephaly (<2 SD)**	4	16
**Macrocephaly (>3 SD)**	4	16
**Fatigability/exercise intolerance**	17	68
**Marked developmental gross motor delays** *	8	32
**Seizures**	5	20
**Movement disorders**	2	8
**Oculomotor abnormality**	4	16
**Ptosis**	2	8
**Sensorineural hearing deficit**	3	12
**Dysarthria**	3	12
**Multiple regressions**	9	36
**Regression(s) after three years of age**	6	24
**At least 1 major clinical finding uncommon in ASD** **	24	96
**At least 1 non-CNS organ system involved**	21	84
**At least 2 non-CNS organ systems involved**	8	32
**At least 1 neurologic finding uncommon in ASD** †	15	60

* Defined as ≥5 SD from mean age at walking [33].

** Does not include multiple gestation birth, prenatal/perinatal complications, GERD, constipation or macrocephaly.

† Does not include fatigability, non-refractory seizures, macrocephaly or regression.

Twenty-one patients had histories of major non-neurological medical problems, most commonly of the gastrointestinal system, with gastroesophageal reflux affecting nine and constipation affecting eight subjects. Seven patients had structural or functional cardiovascular abnormalities. In addition, 17 patients had excessive fatigability or exercise intolerance and several children had abnormal physical exam findings including six with facial dysmorphism, four with microcephaly, four with macrocephaly, and five with growth retardation.

Twelve patients had neurological findings uncommon in ASD apart from microcephaly, including: oculomotor abnormalities (n = 4), sensorineural hearing deficit (n = 3), dysarthria (n = 3), ptosis (n = 2), movement disorders (n = 2), and hypertonia (n = 1). Five patients had seizures with one having medically refractory epilepsy.

Eight patients had markedly delayed early motor milestones. Of 14 patients with regression of previously acquired skills, nine had multiple regressions, six regressed at ages older than three years, and seven had regressions occurring with infections or other metabolic stresses. In six, gross motor skills were lost in addition to language skills. In one case, the timing of regression coincided with a recent vaccination.

Family history data were notable for likely autosomal recessive inheritance in one patient whose parents were first cousins. The family history suggested mitochondrial inheritance in three patients. Three other patients had mothers with features suggesting mitochondrial disease and four patients had sibs with either enzyme-defined mitochondrial disease and/or a clinical history consistent with mitochondrial disease.

### Biochemical and Neuroimaging Data (Table 2; Tables S4 and S5)

Blood lactate was measured in all patients; 19 had at least one increased lactate level, 13 had multiple high levels and 14 had at least one lactate level greater than 3 mmol/L. Of 17 patients who had at least one blood pyruvate determination, nine (53% of those tested) had at least one increased pyruvate level. Plasma amino acids were analyzed for all patients; nine had at least one increased plasma alanine level and four had multiple high alanine levels. The level of AST and/or ALT was increased in 13. One patient had an increased ALT with a normal AST and seven had elevated AST with normal ALT. Eight patients (28%) had increased serum CK levels. Ten patients (42% of those tested) had urinary organic acid abnormalities indicative of mitochondrial dysfunction. The fibroblast lactate/pyruvate ratio was increased for three patients.

**Table 2:** Biochemical and Neuroimaging Data.

	Number abnormal	Number tested	Percent of tested who were abnormal
**Increased blood lactate level**	19	25	76
**Increased blood pyruvate level**	9	17	53
**Increased plasma alanine level**	9	25	36
**Increased serum AST and/or ALT level**	13	25	52
**Increased serum CK level**	8	25	32
**Abnormal urinary organic acid analysis**	10	24	42
**Increased fibroblast lactate:pyruvate ratio**	3	15	20
**Biochemical evidence of mitochondrial disease with any of the above tests**	24	25	96
**Abnormal cranial MRI**	10	21	48
**Increased lactate on cranial MRS**	2	5	40

Twenty-one patients underwent cranial MRI studies; five also had cranial MRS. Ten children had abnormalities on MRI (48% of those imaged), without a single finding predominating, while two patients had increased lactate on MRS (40% of those imaged).

### Histological and Ultrastructural Findings (Table 3)

The most common histological abnormalities in muscle were atrophic or regenerating fibers (n = 8), reduced cytochrome oxidase (COX) staining (n = 6), variation in fiber size (n = 5), and increased myofiber lipid (n = 5). The most common ultrastructural abnormalities were abnormal mitochondrial morphology (n = 4) and increased number of mitochondria (n = 4).

**Table 3:** Muscle Histology and Ultrastructure.

	Number of patients
**Moderate to large variation in fiber size**	5
**Atrophic or regenerating fibers**	8
**Focal inflammation**	4
**Increased myofiber lipid content**	5
**Type I fiber predominance**	1
**COX-negative fibers or reduced COX staining**	6
**Ragged red fibers**	2
**Increased number or subsarcolemmal aggregates of mitochondria**	4
**Mitochondria with abnormal morphology**	4

### Enzyme and Mitochondrial Mutation Data (Table 4; Table S6)

Twenty patients had deficient activity of a respiratory chain complex on tissue ETC or polarographic analysis; three others had phenotypes and biochemical profiles consistent with mitochondrial disease and a sib with less than 20% activity on muscle ETC determination. Specific abnormalities included complex I defect in 16, complex III defect in five, complex II defect in two, and complex IV defect in one. Two patients had mtDNA mutations of likely pathogenicity and four had mtDNA sequence variants of unclear pathogenicity.

**Table 4:** Enzymology and Genetic Data.

	Number of patients	Percent of patients	Mutations
**Complex I defect**	16	64	
**Complex II defect**	2	8	
**Complex III defect**	5	20	
**Complex IV defect**	1	4	
**mtDNA tRNA mutation**	1	4	
**mtDNA sequence variants of probable pathogenicity**	2	8	3397A>G; 4295A>G
**mtDNA sequence variants of unclear pathogenicity**	4	16	3394T>C; 10394C>T; 11809T>C; 11984T>C

## Discussion

The cohort of 25 patients reported here comprises the largest group of individuals with co-occurrence of ASD and defective oxidative phosphorylation reported to date. While previous case reports implicated an association of ASD and mitochondrial dysfunction, it could be argued that this was a chance occurrence in those individuals. Recent epidemiological studies indicate a population prevalence of ASD in about one in 166 children [2] and of mitochondrial disease in about one in 5–10,000 children [30], [31]. These data, and the occurrence of definite oxidative phosphorylation dysfunction in approximately 7% of children with ASD in a population-based cohort [15], provide an epidemiological argument for a non-chance occurrence of ASD and mitochondrial disorders.

Our results indicate diverse and complex developmental, neurological, and medical phenotypes of persons with mitochondrial autism, nearly all of which differ from those of patients with idiopathic ASD. Although many children with ASD exhibit some degree of hypotonia, most attain their early gross motor milestones on time [32]. In contrast, 64% of our patients were delayed in attaining early developmental milestones and 32% were five or more standard deviations later than the mean in walking independently [33]. In addition, although regression has been reported to occur in approximately one third of autistic children, typically before age three years [34], 40% of our patients demonstrated unusual patterns of regression–either repeated regressions, regressions involving losses of gross motor function, and/or regressions after age three years.

Recently, there has been increased concern regarding a possible causative role of vaccinations in autistic children with an underlying mitochondrial cytopathy [35], [36]. For one of our 25 patients, the child's autism/neurodevelopmental deterioration appeared to follow vaccination [12], [36]. Although there may have been a temporal relationship of the events in this case, such timing does not prove causation. That said, there might be no difference between the inflammatory or catabolic stress of vaccinations and that of common childhood diseases, which are known precipitants of mitochondrial regression [37]. Large, population-based studies will be needed to identify a possible relationship of vaccination with autistic regression in persons with mitochondrial cytopathies.

In addition to atypical developmental patterns, non-neurological disorders were nearly universal in our patients. Although medical co-morbidities are not uncommon in individuals with ASD, they are not reported to be present with the high frequency noted here [38]. As in persons with idiopathic ASD, gastrointestinal dysfunction represented the most common non-neurological abnormality in our cohort. However, several of our patients had pancreatic dysfunction or liver disease–gastrointestinal disorders that are rare in persons with ASD [39], [40]. The other organ system dysfunctions in our patients (cardiac, hematological, growth retardation, fatigability) are known manifestations of mitochondrial disease [41]–[43] but are not typical co-morbidities of primary autism [38].

Along with medical co-morbidities, an increased frequency of prenatal and perinatal complications has been reported in children with ASD [44]. However, whether these complications themselves increase the risk for autism or are consequences of an underlying disorder that predisposes to autism is unknown. Our study also shows a high frequency of prenatal/perinatal complications whose relationship to the children's autism is similarly unclear. Of note, an increased incidence of prenatal/perinatal complications has been reported for children with mitochondrial disease [45].

Besides prenatal complications, our data indicate a high frequency of multiple gestation births. The relevance of this finding is unclear but interesting in view of previous controversy about twinning as a risk factor for autism [46].

Also dramatically different from the general ASD population, the sex distribution of the subjects in our cohort is approximately 1∶1. Primary ASD is 3–6 times more common in males than females and this ratio is even higher in PDD-NOS [1], [2]. Since 56% of our patients have PDD-NOS, our finding of a nearly even distribution of males and females is especially notable.

From a biochemical perspective, patients' blood and urine laboratory data revealed marked interindividual variation that did not appear to correspond with specific ETC defects or clinical phenotypes. Significant intraindividual variation was also noted for determinations of blood lactate and pyruvate levels, plasma alanine levels, serum transaminases and CPK, and urinary organic acids, with intermittent normal levels in most cases. The histological and ultrastructural abnormalities noted on muscle biopsies were also varied amongst the subjects of this cohort and similar to results noted in other cohorts of children with mitochondrial electron transport chain disorders [47].

For most individuals with defects of oxidative phosphorylation, the diagnosis is made through ETC determination but an underlying nuclear or mitochondrial mutation usually cannot be identified [43], [48], [49]. The biochemical assessment of mitochondrial disorders, especially ETC enzyme assay, is complex and subject to limitations [49], [50]. Even a clear-cut deficiency of one or more ETC activities *in vitro* does not prove a genetic defect of oxidative phosphorylation because ETC deficiencies can be secondary to other conditions [41], [49]–[51]. The biochemical cut-off to diagnose a deficiency of an ETC varies by laboratory and multiple sets of diagnostic criteria are in use [49]. The criteria used in this study reflect an attempt to be diagnostically conservative. We excluded patients with clearly reduced but >20% ETC activity who lacked a pathogenic mutation even though less restrictive criteria are commonly used.

ETC complex I deficiency was the most prevalent enzyme defect, affecting 64% of our patients, followed by complex III deficiency, affecting 20%. It should be noted, however, that we cannot exclude the possibility that some patients might have biochemically mild forms of multiple ETC deficiencies. The predominance of ETC complex I deficiency is not unexpected and has been noted in cohorts of non-autistic patients with mitochondrial cytopathies [52]. This presumably reflects the large number of genes encoding complex I structure, assembly, and regulation.

Of patients who underwent mitochondrial genomic analysis, two had rare homoplasmic DNA sequence variants of likely pathogenicity: mtDNA 3397A>G and 4295A>G. mtDNA 3397A>G, which replaces methionine with valine in a highly conserved region of the ND1 subunit of complex I, has been implicated in various neurological phenotypes and other conditions [53]–[56]. Our interpretation of its likely pathogenicity is also based on the patient's reduced muscle ETC complex I activity and increased fibroblast lactate:pyruvate ratio. mtDNA 4295A>G occurs in an absolutely evolutionarily conserved sequence of mt tRNA^Ileu^, reduces 3′ tRNAse processing efficiency [57], segregates with multiple disease states [58]–[60], and has been categorized as probably pathogenic [29]. A third patient had a rare sequence variant, 11984T>C. This mutation, reported previously in a child with Leigh syndrome, is likely pathogenic as it is a missense mutation of the ND4 subunit of complex I that replaces a highly conserved tyrosine with histidine that, in turn, is predicted to markedly alter protein structure [61]. Its rarity and the absence of *in vitro* functional studies preclude assignment of probable pathogenicity at this time. For all three patients with these mutations there was intrafamilial phenotypic heterogeneity, as well as differences from phenotypes reported in the literature. Undetected heteroplasmy could explain these differences, but intrafamilial phenotypic variation with homoplasmic mitochondrial mutations has been reported and reasonable explanations proposed [42], [62], [63]. Because of the variability of phenotypes associated with these putative mutations, it is possible that there are important environmental or genetic factors in addition to the mtDNA mutation that contribute to the development of autism in a child with one of these mtDNA mutations. The mutations noted here are different from those described in prior case reports of children with autism and mitochondrial disease [10], [13], [14].

Overall, our results demonstrate substantial clinical heterogeneity of individuals with co-occurring autism and defects of mitochondrial oxidative phosphorylation, nearly all of whom we found to be clinically distinct from children with idiopathic autism. The data do not exclude the possibility of persons with isolated autism having a disorder of oxidative phosphorylation–in fact, one of our patients did not have any major clinical features that distinguished her from typical autism. In addition, it is possible, if not likely, that a still broader clinical, biochemical and genetic spectrum of mitochondrial autism exists.

Finally, data from multiple disciplines, especially research in developmental neurobiology and genetics, point to several underlying pathophysiological mechanisms in autism, including altered neurite morphology, synaptogenesis and cell migration due to abnormalities in distinct ensembles of proteins and pathways [64]. The data reported here, and other cases of mitochondrial autism, argue that defective mitochondrial oxidative phosphorylation is an additional pathogenetic basis for a subset of individuals with autism.

## Supporting Information

Table S1 ASD Diagnosis and Mitochondrial Disease Criteria. PDD-NOS = Pervasive developmental disorder–not otherwise specified, MDC = mitochondrial disease criteria
(0.07 MB DOC)

Table S2 Pertinent Medical History. GERD = gastroesophageal reflux disease, POTS = postural orthostatic tachycardia syndrome, RBBB = right bundle branch block, EF = ejection fraction, LVH = left ventricular hypertrophy
(0.05 MB DOC)

Table S3 Regression History. Blank cells indicate an absence of regression of the type listed in the column
(0.05 MB DOC)

Table S4 Biochemical Data. L:P = lactate to pyruvate ratio, DCA = dicarboxylic acids, EMA = ethylmalonate, 3-MG = 3-methylglutaconate * Value is listed only if level was higher than the upper limit of the reference interval † Patient had one high level but exact value is unknown
(0.06 MB DOC)

Table S5 Neuroimaging. Blank cells indicate that patient did not undergo cranial MRI
(0.04 MB DOC)

Table S6 Enzymology and mtDNA Testing 1 Testing performed at CIDEM Lab, Cleveland 2 Testing performed at Horizon Molecular Medicine LLC, Atlanta A: selected point mutations screened B: mtDNA sequencing C: whole mitochondrial genome scan by dHPLC P: mutation of probable pathogenicity U: mutation of unclear pathogenicity OXPHOS: polarographic determination of mitochondrial oxidative phosphorylation reactions * This patient does not have mitochondrial sequence changes indicative of haplogroup K †ETC complex III, when measured separately, was normal in these patients
(0.06 MB DOC)
