# Supplementary material for: Mitochondrial Disease in Autism Spectrum Disorder Patients: A Cohort Analysis
Source: PLoS One. 2008 Nov 26;3(11):e3815. doi: 10.1371/journal.pone.0003815 (PMC2584230; doi:10.1371/journal.pone.0003815)
Supplement: Table S1 — ASD Diagnosis and Mitochondrial Disease Criteria. PDD-NOS = Pervasive developmental disorder–not otherwise specified, MDC = mitochondrial disease criteria (0.07 MB DOC) [file pone.0003815.s001.doc]

| **Patient number** | **Gender** | **Age (yrs)** | **Autism diagnosis by DSM-IV-TR** | **Modified Walker Criteria** | **MDC criteria** | **MDC score** |
| --- | --- | --- | --- | --- | --- | --- |
| 1 | F | 7 | PDD-NOS | definite | probable | 7 |
| 2 | M | 9 | PDD-NOS | definite | probable | 5 |
| 3 | M | 9 | PDD-NOS | definite | probable | 7 |
| 4 | F | 15 | PDD-NOS | probable | probable | 6 |
| 5 | M | 2 | Autistic Disorder | definite | definite | 8 |
| 6 | F | 16 | PDD-NOS | probable | definite | 9 |
| 7 | M | 20 | Autistic Disorder | definite | definite | 10 |
| 8 | M | 6 | Autistic Disorder | definite | definite | 9 |
| 9 | M | 15 | Autistic Disorder | probable | probable | 5 |
| 10 | F | 4 | Autistic Disorder | definite | definite | 9 |
| 11 | F | 8 | PDD-NOS | definite | definite | 8 |
| 12 | F | 3 | PDD-NOS | definite | probable | 5 |
| 13 | M | 10 | PDD-NOS | definite | probable | 5 |
| 14 | M | 6 | Autistic Disorder | definite | definite | 10 |
| 15 | M | 17 | Autistic Disorder | definite | probable | 5 |
| 16 | F | 14 | PDD-NOS | definite | definite | 8 |
| 17 | M | 10 | Autistic Disorder | probable | probable | 6 |
| 18 | F | 12 | PDD-NOS | definite | definite | 8 |
| 19 | F | 9 | PDD-NOS | definite | definite | 12 |
| 20 | F | 5 | Autistic Disorder | definite | probable | 7 |
| 21 | M | 5 | PDD-NOS | definite | definite | 10 |
| 22 | F | 10 | PDD-NOS | definite | probable | 7 |
| 23 | M | 13 | Autistic Disorder | definite | probable | 6 |
| 24 | M | 11 | PDD-NOS | definite | probable | 5 |
| 25 | F | 3 | Autistic Disorder | definite | probable | 5 |
