# Supplementary material for: Mitochondrial Disease in Autism Spectrum Disorder Patients: A Cohort Analysis
Source: PLoS One. 2008 Nov 26;3(11):e3815. doi: 10.1371/journal.pone.0003815 (PMC2584230; doi:10.1371/journal.pone.0003815)
Supplement: Table S2 — Pertinent Medical History. GERD = gastroesophageal reflux disease, POTS = postural orthostatic tachycardia syndrome, RBBB = right bundle branch block, EF = ejection fraction, LVH = left ventricular hypertrophy (0.05 MB DOC) [file pone.0003815.s002.doc]

| **Patient number** | **Medical history** | **Neurological history** | **Constitutional symptoms** |
| --- | --- | --- | --- |
| 1 |  | dysarthria, loss of bladder & bowel continence | fatigability, frequent infections |
| 2 | GERD |  | fatigability, heat and cold intolerance, frequent infections |
| 3 | cardiac conduction block | unilateral hearing loss | fatigability, heat and cold intolerance, frequent infections |
| 4 | POTS, intermittent proteinuria | macrocephaly, nystagmus, left sensorineural deafness, neonatal hypotonia | fatigability |
| 5 | GERD, chronic diarrhea, RBBB | macrocephaly, hyperopia, esotropia, neonatal hypotonia, ptosis | fatigability, exercise intolerance, frequent infections |
| 6 | GERD, constipation | seizures, Duane retraction syndrome | fatigability, exercise intolerance, muscle cramps |
| 7 | left ventricular dysfunction w/ EF 20%, benign adrenal tumor | parkinsonism, left hemiplegia, dysphagia, seizures in infancy, intention tremor, dysmetria, hyperreflexia | fatigability, muscle cramps |
| 8 | GERD, growth retardation, reactive airway disease, extra-aortic arch | microcephaly |  |
| 9 | constipation, growth retardation | microcephaly, facial droop, bilateral sensorineural hearing loss, strabismus, oromotor dyspraxia | fatigability, heat and cold intolerance |
| 10 | chronic diarrhea, failure to thrive |  | frequent infections |
| 11 | GERD, cyclic vomiting |  | fatigatbility, frequent infections |
| 12 |  |  |  |
| 13 | cryptorchidism, micropenis, constipation | microcephaly |  |
| 14 | neutropenia, fatty liver, recurrent pancreatitis, LVH, RBBB | refractory epilepsy | frequent infections |
| 15 | GERD, constipation | macrocephaly | fatigability, heat intolerance, muscle cramps |
| 16 | growth retardation, constipation | hypertonia, hyperreflexia | fatigability |
| 17 |  | dysarthria |  |
| 18 | GERD, scoliosis, constipation |  | fatigability, heat intolerance, exercise intolerance |
| 19 | growth retardation, hypothyroid | microcephaly, seizures, headaches | fatigability |
| 20 | acute renal failure, constipation |  | exercise intolerance |
| 21 | congenital cardiomyopathy, growth retardation, anemia, thrombocytopenia, RBBB, cardiomegaly | ptosis |  |
| 22 | GERD, constipation | macrocephaly |  |
| 23 | GERD |  | fatigability |
| 24 |  | oromotor dyspraxia, tremor, ataxia, dysarthria | fatigability, exercise intolerance, frequent infections |
| 25 | pancreatic exocrine insufficiency, chronic diarrhea | seizures | fatigability, exercise intolerance, frequent infections |
