# Supplementary material for: Mitochondrial Disease in Autism Spectrum Disorder Patients: A Cohort Analysis
Source: PLoS One. 2008 Nov 26;3(11):e3815. doi: 10.1371/journal.pone.0003815 (PMC2584230; doi:10.1371/journal.pone.0003815)
Supplement: Table S3 — Regression History. Blank cells indicate an absence of regression of the type listed in the column (0.05 MB DOC) [file pone.0003815.s003.doc]

| **Patient number** | **Number of regressions** | **Regression(s) after 3 yrs** | **Regression(s) with catabolic stress** | **Areas of regression(s)** |
| --- | --- | --- | --- | --- |
| 1 | multiple | yes | yes | gross motor, language, social |
| 2 |  |  |  |  |
| 3 | multiple | yes |  | language |
| 4 |  |  |  |  |
| 5 | multiple |  | yes | gross motor, language, social |
| 6 |  |  |  |  |
| 7 | multiple | yes |  | gross motor, language |
| 8 |  |  |  |  |
| 9 |  |  |  |  |
| 10 | one |  |  | gross motor, language, social |
| 11 | multiple | yes | yes | gross motor, language, social |
| 12 |  |  |  |  |
| 13 | one |  |  | language |
| 14 | one |  |  | social |
| 15 |  |  |  |  |
| 16 |  |  |  |  |
| 17 | one |  |  | language, social |
| 18 | multiple | yes | yes | language, social |
| 19 | multiple |  | yes | gross motor, language |
| 20 | multiple | yes | yes | language, social |
| 21 |  |  |  |  |
| 22 |  |  |  |  |
| 23 | one |  |  | language, social |
| 24 |  |  |  |  |
| 25 | multiple |  | yes | language |
