# Supplementary material for: Mitochondrial Disease in Autism Spectrum Disorder Patients: A Cohort Analysis
Source: PLoS One. 2008 Nov 26;3(11):e3815. doi: 10.1371/journal.pone.0003815 (PMC2584230; doi:10.1371/journal.pone.0003815)
Supplement: Table S4 — Biochemical Data. L:P = lactate to pyruvate ratio, DCA = dicarboxylic acids, EMA = ethylmalonate, 3-MG = 3-methylglutaconate * Value is listed only if level was higher than the upper limit of the reference interval † Patient had one high level but exact value is unknown (0.06 MB DOC) [file pone.0003815.s004.doc]

| **Patient number** | **Highest blood lactate level (mmol/L)*** | **Highest blood pyruvate level (mmol/L)*** | **Highest plasma alanine level (μmol/L)*** | **Fibroblast L:P** | **Urinary organic acids** |
| --- | --- | --- | --- | --- | --- |
| 1 |  |  |  |  |  |
| 2 | 2.5 | 0.16 | 610 |  |  |
| 3 | 7.8 | 0.22 |  |  |  |
| 4 |  | 0.13 | 632 |  |  |
| 5 | 5.8 |  | 533 |  | succinate, aconitate, citrate |
| 6 | 3.9 |  |  |  |  |
| 7 |  | 0.07 | 605 |  |  |
| 8 | 2.7 | 0.11 | 478 |  | TCA cycle intermediates, 3-MG |
| 9 | 3.3 |  |  | increased |  |
| 10 | 3.3 |  |  |  | DCA, EMA, methylsuccinate |
| 11 | 3.5 |  |  |  |  |
| 12 | 6.8 | 0.2 |  |  |  |
| 13 | 3.6 |  |  |  | ethylhydracylate |
| 14 |  |  | 558 |  | fumarate, 3-MG, DCA |
| 15 | 7.7 |  |  |  |  |
| 16 | 2.9 |  |  |  | EMA, lactate, glutarate, fumarate, adipic acid, 3-hydroxybutyrate |
| 17 |  | 0.12 | 711 |  |  |
| 18 | 3.8 |  |  | increased | 3-MG |
| 19 | 5.9 |  |  | increased | 2-hydroxyglutarate, lactate |
| 20 | 5.9 | 0.18 |  |  | 3-MG |
| 21 | 4.3 |  | † |  | glutarate, lactate, fumarate, methylsuccinate, DCA, EMA |
| 22 | 2.9 | 0.17 | 524 |  |  |
| 23 | 4.7 |  |  |  |  |
| 24 | 2.3 |  |  |  |  |
| 25 |  |  |  |  |  |
