# Supplementary material for: Mitochondrial Disease in Autism Spectrum Disorder Patients: A Cohort Analysis
Source: PLoS One. 2008 Nov 26;3(11):e3815. doi: 10.1371/journal.pone.0003815 (PMC2584230; doi:10.1371/journal.pone.0003815)
Supplement: Table S5 — Neuroimaging. Blank cells indicate that patient did not undergo cranial MRI (0.04 MB DOC) [file pone.0003815.s005.doc]

| **Patient number** | **Most recent MRI/MRS** |
| --- | --- |
| 1 | normal (5 yrs) |
| 2 | normal (2 yrs) |
| 3 |  |
| 4 | normal (10 yrs) |
| 5 | normal (8 mos) |
| 6 | periventricular white matter abnormalities / normal MRS (16 yrs) |
| 7 | mild central white matter volume loss (16 yrs) |
| 8 | abnormal basal ganglia (9 mos) |
| 9 | normal (12 yrs) |
| 10 | normal (2 yrs) |
| 11 | normal (2 yrs) |
| 12 |  |
| 13 | mildly prominent ventricles, enlarged subarachnoid spaces, T2 hyperintensity of parietal subcortical white matter (7 mos) |
| 14 | increased T2 signal right thalamus / normal MRS (4 yrs) |
| 15 | normal (7 yrs) |
| 16 | mildly prominent ventricles on MRI / increased lactate in dentate nuclei on MRS (5 yrs) |
| 17 |  |
| 18 | increased T2 signal right periatrial white matter (3 yrs) |
| 19 | normal MRI / increased lactate diffusely especially in cerebellar gray matter on MRS (9 mos) |
| 20 |  |
| 21 | periventricular white matter abnormalities (2 yrs) |
| 22 | mildly prominent ventricles, mildly increased signal periventricular region (2 yrs) |
| 23 | normal (7 yrs) |
| 24 | increased T2 signal right amygdala (7 yrs) |
| 25 | normal (2 yrs) |
