# Supplementary material for: Mitochondrial Disease in Autism Spectrum Disorder Patients: A Cohort Analysis
Source: PLoS One. 2008 Nov 26;3(11):e3815. doi: 10.1371/journal.pone.0003815 (PMC2584230; doi:10.1371/journal.pone.0003815)
Supplement: Table S6 — Enzymology and mtDNA Testing 1 Testing performed at CIDEM Lab, Cleveland 2 Testing performed at Horizon Molecular Medicine LLC, Atlanta A: selected point mutations screened B: mtDNA sequencing C: whole mitochondrial genome scan by dHPLC P: mutation of probable pathogenicity U: mutation of unclear pathogenicity OXPHOS: polarographic determination of mitochondrial oxidative phosphorylation reactions * This patient does not have mitochondrial sequence changes indicative of haplogroup K †ETC complex III, when measured separately, was normal in these patients (0.06 MB DOC) [file pone.0003815.s006.doc]

| **Patient number** | **Final diagnosis** | **Percent enzyme activity** | **Diagnostic enzymology** | **mtDNA testing** | **Relevant mutations found** |
| --- | --- | --- | --- | --- | --- |
| 1 | complex I | I+III (11%)^†^ | Muscle homogenate^1^ | A |  |
| 2 | complex I | brother: I (0%) | Isolated muscle mito^2^ | A |  |
| 3 | complex I | I (0%) | Isolated muscle mito^2^ | A |  |
| 4 | mtDNA tRNA^Ile^ mutation |  |  | C | 4295A>G (P)* |
| 5 | complex I | I (0%) | Isolated muscle mito^2^ | C |  |
| 6 | complex II |  | Muscle OXPHOS^1^ | A |  |
| 7 | complex III | I + III (20%); III (20%) | Isolated muscle mito^1^; skin^1^ | C |  |
| 8 | complex I | I (0%) | Isolated muscle mito^2^ | A |  |
| 9 | mtDNA mutation in complex I | I (26%) |  | A, C | 3397A>G (P) |
| 10 | complex I | I (0%), I+III (14%) | Isolated muscle mito^2^ | A |  |
| 11 | complex I | sister: I (0%) | Isolated muscle mito^2^ | sister = A |  |
| 12 | complex I | I (0%), I+III (10%) | Isolated muscle mito^2^ | A |  |
| 13 | complex I | I (0%) | Muscle homogenate^1^ | A |  |
| 14 | complex IV |  | Muscle OXPHOS^1^ | A, C | 11809T>C (U) |
| 15 | complex I | I+III (10%)^†^ | Muscle homogenate^1^ | A, C |  |
| 16 | complex I | I (0%) | Isolated muscle mito^2^ | B |  |
| 17 | complex III |  | Muscle OXPHOS^1^ | C |  |
| 18 | complex II | II+III (18%), II (32%) | Muscle homogenate^1^ | A |  |
| 19 | complex III | III (18%) | Isolated muscle mito^1^ |  |  |
| 20 | complex III | I+III (15%), III (15%); III (28%) | Muscle homogenate^1^; skin^1^ | C |  |
| 21 | complex III | III (14%) | Liver homogenate^1^ |  |  |
| 22 | complex I | I+III (7%)^†^ | Muscle homogenate^1^ | A | 3394T>C (U) |
| 23 | complex I | sister: I (0%), I+III (15%) | Isolated muscle mito^2^ | A, C | 11984T>C (U) |
| 24 | complex I | I (0%), I+III (19%) | Isolated muscle mito^2^ | A, C |  |
| 25 | complex I | I (20%) | Isolated muscle mito^2^ | A, C | 10394C>T (U) |
